# Perceived health effects of vaping among Hungarian adult e-cigarette-only and dual users: a cross-sectional internet survey

**DOI:** 10.1186/s12889-019-6629-0

**Published:** 2019-03-13

**Authors:** Lilla Abafalvi, Melinda Pénzes, Róbert Urbán, Kristie L. Foley, Réka Kaán, Barbara Kispélyi, Péter Hermann

**Affiliations:** 1 Department of Prosthodontics, Faculty of Dentistry, Semmelweis University, Üllői út 26, Budapest, H-1085 Hungary; 2 Institute of Public Health, Faculty of Medicine, Semmelweis University, Üllői út 26, Budapest, H-1085 Hungary; 3 Institute of Psychology, Eötvös Loránd University, Izabella utca 46, Budapest, H-1064 Hungary; 4 Department of Implementation Science, Wake Forest University School of Medicine, Medical Center Boulevard, Winston Salem, NC 27157 USA

**Keywords:** E-cigarette, Vaping, Adverse event, Smoking, Health, Health consequence

## Abstract

**Background:**

Knowledge about the health effects of e-cigarette use (or vaping) among past and current combustible cigarette users is limited. Several studies have assessed vaping-related adverse events (AEs) and beneficial health effects, however, most studies focused on AEs in general and examined only a few physiological changes that vapers experience. This study aims to explore self-reported AEs and perceived health changes due to e-cigarette use among Hungarian adult e-cigarette-only users (former smokers who switched completely to e-cigarette use) and dual users (smokers who use e-cigarettes and combustible tobacco cigarettes concomitantly).

**Methods:**

A cross-sectional, web-based survey of 1042 adult Hungarian e-cigarette users was conducted in 2015. Participants reported AEs and changes in physiological functions since they switched from smoking to e-cigarette use or while dually using e-cigarettes and combustible cigarettes. Confirmatory factor analysis with covariates was applied to explain perceived health changes due to e-cigarette-only use and dual use.

**Results:**

Dual users (17.6%) were significantly more likely to report AEs of vaping than e-cigarette-only users (26.2% vs. 11.8%, *p* < 0.001). Experiencing health improvements were significantly more likely among e-cigarette-only users than for dual users for all surveyed physiological functions. E-cigarette-only users reported larger effects of vaping on sensory, physical functioning, and mental health factors compared to dual users. Self-reported changes in sensory and physical functioning were significantly higher among individuals using e-cigarettes more than a year and people who were past heavy smokers (smoked ≥20 cigarettes per day). Gender was related to sensory improvement only; males reported greater improvement than females.

**Conclusions:**

The majority of e-cigarette-only users reported more perceived beneficial changes in physiological functions and fewer AEs than dual users. Perceived short-term benefits of e-cigarette use may reinforce users despite the uncertainty of long-term health consequences. Health professionals should provide balanced information regarding the possible short- and long-term positive and negative health effects of e-cigarette use during consultations with patients.

**Electronic supplementary material:**

The online version of this article (10.1186/s12889-019-6629-0) contains supplementary material, which is available to authorized users.

## Background

Electronic cigarettes (e-cigarettes) are rapidly evolving products with a wide range of design and engineering variations. The basic function of all e-cigarettes is to generate a heated aerosol typically containing nicotine, that is inhaled through a mouthpiece [1, 2]. Some studies have shown that e-cigarettes may emit substantially less toxic substances than combustible cigarettes [3, 4], although others have found higher metal and silicate concentration in the aerosol compared to combustible cigarette smoke [5], while nicotine intake from advanced generation devices is similar to that of tobacco cigarettes [1].

There is an ongoing debate on the potential harms or benefits of e-cigarettes in the public health community. Advocates of e-cigarettes emphasize the harm reduction potential of these products as substitutes for combustible tobacco among persons trying to quit or reducing the amount of cigarette smoking (aka dual users). However, dual use will undermine the potential harm reduction due to the known short- and long-term health effects of smoking [6]. On the other hand, opponents argue that there are unknown long-term risks of e-cigarette use (or vaping). These include the potential for negative health effects, e-cigarettes serving as a gateway to traditional tobacco use among youth, and a decreased likelihood of conventional cigarette smoking cessation [6, 7]. The overall population health effect of e-cigarette use remains uncertain. Some data demonstrate a favorable public health impact of switching from cigarettes to e-cigarette use [8], while others are more cautious, emphasizing the uncertainties of health harm, instigating tobacco initiation among youth, and maintaining addiction among adults [9].

Despite the debate and growing perceptions that e-cigarettes are harmful, experimentation with e-cigarettes is increasing in the European Union [10]. In Hungary, there are limited data about e-cigarette use among adults. In 2017, 27% of Hungarian persons 15 years-old and older were current smokers, and 9% had ever tried an e-cigarette [11]. A national study conducted among Hungarian young adults (mean age 21.7 years) found a 24.9% ever use rate, but low current (past 30-day) use rate of e-cigarettes (0.6%) [12].

Current knowledge about both self-reported and objectively measured health effects of e-cigarette use among past combustible cigarette users is limited. Among the scant literature, most health effects were measured by self-report rather than objectively measured. In recent years, several studies have assessed vaping-related adverse events (AEs) and beneficial health effects. However, most studies focused on AEs in general and consistently examined only a few physiological changes that e-cigarette users experience. Of studies indicating no conflicts of interest regarding support from e-cigarette manufacturers or lobby, 84.2% of Dutch vapers reported health improvements since e-cigarette use initiation [13], while 84% of German ex-smoker vapers indicated an overall feeling of living healthier [14]. Likewise, 71% of Italian smokers who switched from cigarettes to e-cigarettes experienced general health improvements after three months [15]. It is unclear if these perceived health improvements are due to placebo effect, reflect expectancies driven by e-cigarette marketing, or are true physiological changes. Improvements in specific physiological conditions in persons who replace combustible cigarettes with e-cigarettes are less well-documented. Besides respiratory, cardiovascular and sensory changes associated with vaping, other physiological changes have been observed, for instance, in periodontal health, appetite, quality of sleep, mood, and sexual performance [2, 13–18]. Although hundreds of vaping-related AEs have been registered [16], e-cigarette users report mostly mild AEs, including mouth/throat irritation, dry cough, dry mouth, dizziness and headache [17, 19, 20].

This study aims to explore self-reported health effects of vaping among Hungarian adult e-cigarette-only users (former smokers who switched completely to e-cigarettes) and dual users (smokers who use e-cigarettes and combustible tobacco cigarettes concomitantly). Our objectives were (1) to describe characteristics of Hungarian adult e-cigarette-only and dual users, (2) to assess self-reported AEs and changes in physiological functions after e-cigarette use initiation among e-cigarette-only and dual users, and (3) to explain perceived health changes due to e-cigarette use.

## Methods

### Participants and procedure

A cross-sectional online survey was conducted among adult (age 18+) Hungarian e-cigarette users in September–December, 2015. The convenience sample was obtained by posting the survey on Hungarian e-cigarette forum websites (www.ecigiforum.com, www.ecigiblog.blog.hu, www.ecigaretta.eu) and an e-cigarette webshop (www.sbcig.com) inviting website visitors to participate. After reading the description of the study, participants consented to participate by voluntarily answering the survey questions. A lottery-based incentive was offered after two months to increase participation. 800 participants completed the survey anonymously without the incentive, and 784 completed the survey after the incentive was introduced, indicating their e-mail address to participate in the lottery. The study was approved by the Institutional Review Board of Semmelweis University, Budapest, Hungary.

Of the 1584 initial respondents, we excluded the followings: those who were < 18-year-old (*n* = 4); had never smoked (*n* = 22); did not respond whether they used e-cigarette and/or combustible cigarette (*n* = 63); and responded inconsistently to questions assessing e-cigarette-only and dual use (*n* = 40). Since we did not have access to respondents’ internet protocol (IP) address to exclude multiple responses from the same participants, we searched for duplicate cases (*n* = 413) using all socio-demographic variables (gender, age, education level, type of settlement, income level). We applied an a priori decision rule that only the first case of potential duplicate respondents was included in the final analytical sample. As a result, 1042 unique respondents who ever smoked and were current e-cigarette users (only or dual users) were included in the study.

### Measures

The questionnaire consisted of seven parts: (1) socio-demographic characteristics of respondents, (2) e-cigarette use, (3) perceived harm of e-cigarette use, (4) combustible cigarette use, (5) oral hygiene, (6) vaping-related adverse events, and (7) changes in physiological functions (see Additional file 1 for full questionnaire). The following variables were included in the current study:

*Socio-demographic* data were collected on gender, age (range 18–75, mean age 38.9 [SD = 11.8]), and education (technical school or less – without graduation certificate, high school or vocational school – with graduation certificate, and college or university).

*E-cigarette-only* versus *dual use* was assessed by a question “Do you use e-cigarette or combustible cigarette?” (combustible cigarettes only, e-cigarettes only, both of them). Only persons who were e-cigarette-only users and dual users were included in the study.

*Past combustible cigarette use* was measured by the number of tobacco cigarettes smoked per day before initiating e-cigarette use. Response options were categorized into: ≤10 CPD – light smoker, 11–19 CPD – moderate smoker, ≥20 CPD – heavy smoker.

*Current e-cigarette use characteristics* variables included in this study were (1) time since respondent started using e-cigarettes (< 6 month ago, 6–12 months ago, 1–2 years ago, > 2 years ago), (2) frequency of e-cigarette use per day (non-daily, 1–10 times a day, 11–19 times a day, ≥20 times a day), and (3) nicotine concentration of the e-liquid (0 mg/ml – 18 mg/ml or more) (Table 1).

**Table 1:** Descriptive characteristics of the sample by vaping status

Variable	Total, n (%) 1042 (100)	E-cigarette-only user, n (%) 859 (82.4)	Dual user, n (%) 183 (17.6)	*p*-value
Gender				
Male	859 (82.9)	726 (85.0)	133 (73.1)	< 0.001
Female	177 (17.1)	128 (15.0)	49 (26.9)	
Age (years)				
Mean (SD)	38.9 (11.8)	38.8 (11.7)	39.0 (12.6)	0.843
Education				
Technical school or less (without graduation certificate)	235 (24.6)	200 (25.4)	35 (21.0)	0.120
High school or vocational school (with graduation certificate)	416 (43.6)	348 (44.2)	68 (40.7)	
College or university	303 (31.8)	239 (30.4)	64 (38.3)	
Time since started using e-cigarette				
Less than 6 months ago	195 (19.0)	147 (17.3)	48 (26.7)	0.007
6–12 months	192 (18.7)	155 (18.3)	37 (20.6)	
1–2 years	219 (21.3)	181 (21.3)	38 (21.1)	
More than 2 years ago	423 (41.1)	366 (43.1)	57 (31.7)	
Frequency of e-cigarette use				
Non-daily	21 (2.0)	8 (0.9)	13 (7.2)	< 0.001
1–10 times a day	103 (10.0)	76 (9.0)	27 (15.0)	
11–19 times a day	234 (22.8)	184 (21.7)	50 (27.8)	
≥ 20 times a day	668 (65.1)	578 (68.3)	90 (50.0)	
Combustible cigarettes smoked per day (before started using e-cigarette)				
≤ 10 cigarettes per day	101 (9.7)	82 (9.5)	19 (10.7)	0.468
11–19 cigarettes per day	307 (29.6)	249 (29.0)	58 (32.8)	
≥ 20 cigarettes per day	628 (60.6)	528 (61.5)	100 (56.5)	
Nicotine concentration of e-liquid				
0 mg	77 (7.5)	60 (7.0)	17 (9.4)	0.009
1–6 mg	514 (49.8)	444 (52.1)	70 (38.7)	
7–12 mg	349 (33.8)	272 (31.9)	77 (42.5)	
≥ 13 mg	93 (9.0)	76 (8.9)	17 (9.4)	

Note: Some categories do not add to the total due to missing responses

*Vaping-related adverse events* were assessed in a question where participants could indicate (yes/no) if they had experienced any of 14 AEs (Table 2) [17]. Any AEs variable was computed based on responses for the 14 listed conditions (no AEs/any AEs).

**Table 2:** Prevalence of vaping-related adverse events and perceived improvement in physiological functions

Variable	Total, n (%) 1042 (100)	E-cigarette-only user, n (%) 859 (82.4)	Dual user, n (%) 183 (17.6)	*p*-value
Adverse events				
Sore/dry mouth and throat	39 (3.7)	30 (3.5)	9 (4.9)	0.356
Cough	42 (4.0)	18 (2.1)	24 (13.1)	< 0.001
Mouth or tongue sores/inflammation	30 (2.9)	19 (2.2)	11 (6.0)	0.005
Gingivitis, gum bleeding	34 (3.3)	27 (3.1)	7 (3.8)	0.637
Headache	18 (1.7)	13 (1.5)	5 (2.7)	0.251
Dizziness	10 (1.0)	5 (0.6)	5 (2.7)	0.007
Heart palpitation	14 (1.3)	8 (0.9)	6 (3.3)	0.012
Breathing difficulties	10 (1.0)	6 (0.7)	4 (2.2)	0.061
Chest pain	7 (0.7)	4 (0.5)	3 (1.6)	0.078
Sleepiness	3 (0.3)	2 (0.2)	1 (0.5)	0.472
Sleeplessness	3 (0.3)	2 (0.2)	1 (0.5)	0.472
Allergy	2 (0.2)	2 (0.2)	0 (0.0)	0.514
Black tongue	1 (0.1)	1 (0.1)	0 (0.0)	0.644
Nose bleeding	1 (0.1)	1 (0.1)	0 (0.0)	0.644
Any adverse event	149 (14.3)	101 (11.8)	48 (26.2)	< 0.001
Physiological function				
Breathing	930 (90.1)	790 (92.7)	140 (77.8)	< 0.001
Smell	890 (86.1)	767 (90.0)	123 (67.6)	< 0.001
Taste	864 (83.6)	744 (87.2)	120 (66.7)	< 0.001
Physical status in general	833 (80.9)	715 (84.1)	118 (65.6)	< 0.001
Stamina	800 (77.7)	697 (82.0)	103 (57.5)	< 0.001
Mood	524 (51.1)	461 (54.5)	63 (35.2)	< 0.001
Quality of sleep	469 (45.8)	406 (48.1)	63 (34.8)	0.001
Appetite	412 (40.4)	355 (42.2)	57 (31.7)	0.009
Sexual performance	351 (34.4)	306 (36.3)	45 (25.3)	0.005
Memory	282 (27.7)	246 (29.2)	36 (20.3)	0.016

*Changes in physiological functions* were measured by a question listing 10 physiological functions (Table 2) and respondents were asked if they experienced worsening, no change or improvement of each listed health conditions since they initiated e-cigarette use [17]. Response options were collapsed into a binary variable (worsened/no change vs. improved categories) due to low frequencies (0.0–0.6%) of worsened categories of physiological functions variables in the sample.

### Statistical analysis

Descriptive characteristics of the sample, self-reported AEs and changes in physiological functions by current e-cigarette use status were assessed in Pearson’s chi-square test using SPSS version 24.0. Odds of physiological function improvement were tested among e-cigarette-only users compared to dual users with binary logistic regression analyses. The covariances among physiological functions made it necessary to identify the latent factors of health improvement with confirmatory factor analysis (see Brown [21]). Exploratory factor analysis (EFA) and confirmatory factor analysis (CFA) identify the latent variables behind the different groups of physiological functions, therefore confirmatory factor analysis with covariates (CFA with covariates) was applied to explain perceived improvements in health due to e-cigarette use using Mplus 8.0 [22]. CFA includes first testing the measurement model of improvements. Theoretically, we propose three different aspects of improvements including sensory improvement, physical functioning, and mental health improvement. After supporting this measurement model of health improvements, we introduced the structural components of the model, therefore CFA with covariates model can estimate the effect of indicators on latent variables at the same time when direct effects of grouping variables or other continuous variables on the latent variables are also included.

## Results

Compared to dual users (17.6%), e-cigarette-only users (82.4%) were more likely to be male and started using e-cigarettes more than two years prior to the survey (Table 1). The majority of e-cigarette-only users reported vaping more than 20 times a day while only half of dual users responded similarly. Using low nicotine concentration e-liquid (1–6 mg) was reported by 52.1% of e-cigarette-only users, while similar proportion of dual users used medium (7–12 mg) or high (≥13 mg) nicotine concentration e-liquid. There were no significant differences by mean age, educational level, or combustible cigarettes smoked per day before initiating e-cigarette use between the two groups, however, 60.1% of the whole sample was heavy smoker while 9.7% was light smoker.

Any AEs were reported by 14.3% of the sample and significantly more likely among dual versus e-cigarette-only users (26.2% vs. 11.8%, *p* < 0.001). The majority of respondents reported ≤2 AEs (94.0%). The most common AE was cough among dual users and sore/dry mouth and throat among e-cigarette-only users (Table 2). Mouth and throat-related AEs were commonly reported by e-cigarette-only users while dual users indicated mostly cough and oral cavity AEs. Significantly more dual users experienced cough, mouth or tongue sores/inflammation, dizziness, and heart palpitation than e-cigarette-only users.

The majority of the sample reported better breathing, olfactory and gustatory senses since initiating e-cigarette use (Table 2), while improvement in sexual performance and memory were reported less frequently. About 90%-of e-cigarette-only users experienced better breathing, sense of smell and taste while a bit more than two-thirds of dual users did so. Improved mood, sleep quality and appetite were reported by about half of e-cigarette-only users compared to one-third of dual users. About one-third of e-cigarette-only users and 20–25% of dual users indicated improvements in sexual performance and memory. Experiencing health improvements were significantly more likely among e-cigarette-only users than for dual users for all surveyed physiological functions.

### Predictors of health improvements due to e-cigarette use

We performed a series of binary logistic regression analyses to compare the odds of health improvements in both types of users. The unadjusted and adjusted odds ratios are presented in Table 3. Compared to dual users, e-cigarette-only users had more than three times higher odds perceiving improvement in smelling, breathing, tasting and stamina; and reported about two times higher odds improvement in physical status, mood and sleep quality. There were no significant differences in the odds of improvement in appetite, sexual performance and memory between e-cigarette-only users and dual users.

**Table 3:** Perceived improvement in physiological functions in e-cigarette-only users and dual users: binary logistic regression analyses

Variable	OR	[95%CI]	OR_adjusted_^a^	[95%CI]
Breathing	**3.64**	**[2.35–5.63]**	**3.39**	**[2.15–5.33]**
Smell	**4.33**	**[2.95–6.35]**	**4.12**	**[2.77–6.12]**
Taste	**3.41**	**[2.36–4.94]**	**3.21**	**[2.18–4.72]**
Physical status in general	**3.36**	**[2.38–4.74]**	**2.28**	**[1.56–3.32]**
Stamina	**3.36**	**[2.38–4.74]**	**3.11**	**[2.17–4.45]**
Mood	**2.21**	**[1.58–3.08]**	**2.09**	**[1.48–2.96]**
Quality of sleep	**1.74**	**[1.24–2.43]**	**1.70**	**[1.21–2.41]**
Appetite	1.58	[1.12–2.20]	1.57	[1.09–2.25]
Sexual performance	1.68	[1.17–2.43]	1.68	[1.14–2.48]
Memory	1.62	[1.09–2.40]	1.59	[1.06–2.40]

*Note:* Reference group is dual users. ^a^: adjusted for age, gender, and duration of e-cigarette use. All ORs are significant at least at *p* < 0.050. Bolded ORs remained significant after Bonferroni correction for multiple testing. The adjusted level of significance after Bonferroni correction is *p* < 0.005

**Fig. 1:**
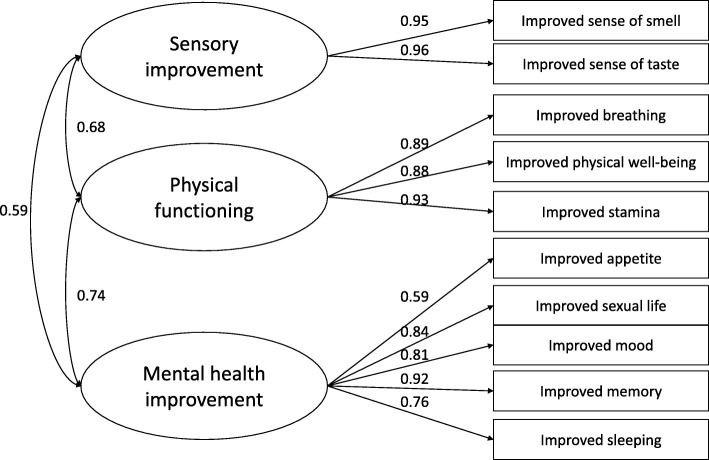
Measurement model of health changes due to e-cigarette use. Standardized factor loadings are presented

We also performed an exploratory factor analysis (EFA) followed by a confirmatory factor analysis (CFA) to estimate the measurement model of improvement in health. We supported three main factors of physiological functions (Fig. 1). The details of this psychometric analysis are available in Additional file 2. The standardized regression coefficients of CFA with covariates are presented in Table 4. Compared to dual users, e-cigarette-only users reported greater improvement in all three dimensions. Improvements in sensory and physical functioning were significantly higher among individuals using e-cigarettes more than a year and people who used to smoke ≥20 CPD, however the size of the difference was highest in sensory improvement factors for the latter variable. Gender was related to sensory improvement only; males reported greater improvements than females.

**Table 4:** Predictors of perceived health improvements since initiating e-cigarette use: confirmatory factor analysis with covariates model

Variable	Sensory improvement	Physical functioning	Mental health improvement
Gender	**−0.32**	−0.12	−0.06
Dual users	**−0.56**	**−0.50**	**−0.29**
Age	−0.01	**−0.01**	**−0.01**
Any adverse event due to e-cigarette use	**−0.58**	**−0.55**	**−0.25**
Educational level	0.07	− 0.08	− 0.09
Duration of e-cigarette use	**0.23**	**0.24**	0.12
Intensity of smoking before e-cigarette use	**0.33**	**0.26**	0.16
High nicotine content of e-liquid	0.14	−0.08	0.06
*R* ^*2*^	16.7%	13.6%	4.2%

Note: Partial regression coefficients. Bolded coefficients are significant at *p* < 0.05. Gender (0: Males, 1: Females). Dual users (0: e-cigarette-only users, 1: Dual users). Age is continuous variable. Any adverse event due to e-cigarette use (0: No adverse event, 1: Report of any adverse event). Educational level (0: Lower than college level education, 1: College or higher level of education). Duration of e-cigarette use (0: A year or less, 1: Longer than a year). Intensity of smoking before e-cigarette use (0: ≤19 cigarettes per day, 1: ≥20 cigarettes per day). High nicotine content of e-liquid (0: ≤6 mg or nicotine-free, 1: ≥7 mg nicotine)

## Discussion

Our study found that e-cigarette-only users report more perceived improvement in health and less adverse health effects of vaping than dual users. The majority of e-cigarette-only users reported better respiratory and sensory functions and indicated only minor AEs since they started using e-cigarettes. However, dual users were probably under-represented while satisfied e-cigarette-only users were over-represented, therefore, our results may overestimate perceived benefits of vaping [14, 23]. Furthermore, the majority of respondents were heavy smoker before e-cigarette use initiation, and only about 10% were light smoker. To our knowledge, this is the first study to explore different dimensions of perceived health changes due to e-cigarette use in a mostly past heavy smoker sample.

Improved olfactory and gustatory functions, both sensory improvements, were frequently reported benefits of vaping in both independent and sponsored studies [13, 15, 17, 23]. High intensity past tobacco smoking and longer e-cigarette use history predicted greater sensory improvements, but only among males. Olfactory dysfunction is more likely among current smokers compared to never smokers, and reduced olfactory ability depends on tobacco smoke dose-duration [24]. Furthermore, smoking-related olfactory dysfunction is reversible due to reversible squamous metaplasia of the olfactory mucosa [24]. Gustatory dysfunction may rather reflect olfactory dysfunction [25], which can explain combined improvement of taste and smell sensation after smoking cessation. Lower exposure to potentially toxic substances from e-cigarettes compared to combustible cigarettes [3] might have a similar but likely weaker effect on the olfactory mucosa similar to cessation, which leads to the regeneration of squamous metaplasia within six months [24].

Improved breathing, general physical status and stamina were correlated with past combustible cigarette smoking habits and e-cigarette use duration. The most commonly reported improvement among these physiological functions was breathing in our whole sample, consistent with studies of Dutch vapers, and a large international sample of a sponsored study involving more than 19,000 e-cigarette users where almost 90% of respondents reported better breathing due to vaping [13, 17, 23]. However, studies investigating respiratory outcomes of e-cigarette use are contradictory. Some studies have demonstrated that short-term e-cigarette use has an acute negative effect on the respiratory system, like impaired lung function and increased airway resistance both among healthy smokers and nonsmokers, and smokers with asthma or COPD [20, 26]. In contrast, others reported no significant short-term changes in lung function parameters among healthy smokers switching to e-cigarettes [18, 27], although studies sponsored by e-cigarette manufacturers and merchants found improvements in spirometric indices and respiratory symptoms on long-term [28, 29]. A large proportion of Hungarian e-cigarette-only users indicated improvement in physical status and endurance similarly to other studies [13–15, 17, 23]. These positive changes may be associated with perceived improvements in respiratory health.

E-cigarette-only users experienced significantly greater improvements in mental health. They reported greater improvements in their mood and quality of sleep compared to a large international study [17] and a smaller one from the Netherlands [13]. However, a study by Adriaens et al. (2017) did not detect significant differences between dual users and switchers (smokers who completely switched to e-cigarette use) in moderate improvement of mood and sleep quality [23]. Possible explanations for mood improvement could be positive expectancies towards e-cigarette use [30], satisfying control on nicotine delivery by advanced generation e-cigarettes [1], perceived supervision of withdrawal symptoms [31], and self-efficacy to change tobacco smoker identity toward a perceived healthier e-cigarette user identity. Adult smokers and former smokers with mental health conditions seem to be more susceptible to trying e-cigarettes and to be current vapers as they perceive them an appealing and less harmful substitutes for conventional cigarettes [32]. Improvements in mood and psychological quality of life as well as perceived better respiratory function might explain positive changes in sleep quality. Nevertheless, a study examining online e-cigarette forum posts related to positive and negative health effects of e-cigarette use explored more negative complaints than positive changes of sleep disorders [16].

Positive changes in memory were reported by a minority of our sample similarly to another study [17]. A previous research explored that memory improved only among individuals using nicotinic e-cigarettes presumably because of impaired memory during smoking abstinence that was reversed by nicotinic e-cigarettes [31].

Sexual performance has rarely been investigated as it pertains to e-cigarette use. Approximately one third of e-cigarette-only users in our sample agreed that their sexual performance improved since they initiated vaping, similarly to an international study which found 28.9% improvement rate [17]. This association is suspected to be complex. On the one hand, nicotine may cause acute vasospasm while other components of tobacco smoke are atherogenic on a dose-response manner both in the male and female genital tract [33, 34]. If e-cigarettes deliver much less toxicants than conventional cigarettes [3], but nicotine delivery by advanced generation devices are as effective as by conventional cigarettes [1], improvement in sexual performance might be expected in individuals with shorter duration and less intense lifetime smoking. On the other hand, better-functioning respiratory and sensory systems may positively influence sexual performance of former smokers switching to e-cigarettes.

Almost two-thirds of dual users and more than half of e-cigarette-only users did not report improved appetite consistent with a few other studies detecting greater improvement in appetite among switchers compared to dual users [17, 23]. It is suspected that both nicotinic and nicotine-free e-cigarettes may prevent weight gain through influencing body metabolism by nicotine and other e-liquid constituents, and providing an alternative activity to eating by replacing high calorie foods with sensory experiences like desirable taste and smell of the vapor [35, 36]. A recent study explored that the popular vanilla-flavored e-liquid was associated with vaping to lose/control weight as it may serve as a distractor from or substitute for high calorie foods [35]. Carbohydrate intake increases after cessation because former smokers perceive the sweet taste more pleasant than smokers possibly due to the activation of central reward centers during nicotine withdrawal [37]. E-cigarettes may have the potential to support and sustain quitting by reducing appetite and weight gain, but in contrast they may also promote the initiation of e-cigarette use for appetite/weight control purposes among non-tobacco users [36].

In our analysis, we also identified three main dimensions of perceived health improvements attributed to e-cigarette use. Sensory improvement included improved smell and taste. The improvement in physical functioning encompassed improved breathing, physical well-being and improved stamina. Finally, the mental health improvement factor incorporated the improved appetite, sexual life, mood, memory and sleeping. We also noted that dual users reported much lower degree of improvement in all three dimensions than e-cigarette-only users. This result highlights that greater perceived health improvements are related with the complete cessation of regular cigarette use. Longer duration of e-cigarette use was associated with higher score on sensory improvement and physical functioning. Interestingly, intensity of smoking before e-cigarette use was also associated with greater improvement in sensory and physical functioning dimensions suggesting that heavy smokers compared to light smokers may gain much more from switching to e-cigarette from combustible cigarette. Furthermore, our study provides a useful self-report tool to measure perceived health effects due to vaping for future studies.

Regarding AEs, mouth and throat, respiratory and neurological symptoms were the most frequently reported by a minority of Hungarian vapers parallel with other studies indicating similar side-effect patterns [16, 17, 19, 20, 23, 27]. Possible explanations of mouth and throat symptoms are multiple. Longer and harder puffing with stronger vacuum is necessary for vaping than for conventional cigarette smoking to produce aerosol [38]. Vacuum generation might involve the tongue, the palate and also the bucca, therefore, these oral regions may be exposed more directly to the vapor [39]. Moreover, glycerin, propylene-glycol, and e-liquid flavorings might form thermal degradation byproducts during vapor generation which may be also a cause of tongue and/or buccal pain as well as mouth and throat irritation [19, 39]. Finally, inhalation of e-cigarette aerosol elements like silicon, sulfur, calcium, titanium, and lithium are associated with throat irritation [5]. Gingivitis and gum bleeding were experienced by our respondents like in previous studies [17, 40]. It is suspected that tobacco smoke-generated vascular changes in the gingiva resolves similarly during vaping and smoking cessation, that is, inflammatory response increases and vasoconstriction decreases in the gingiva [40].

Among AEs, cough was more frequently reported especially by dual users than breathing difficulties. However, cough was less commonly mentioned in our sample compared to previous studies reporting frequencies between 12.8–69.0% [17, 18, 20, 28]. Possible mechanism of cough is a vagal mediated protective reflex generated by inhaled irritants from the vapor like propylene-glycol and/or flavorings [20]. Experiencing neurological AEs (e.g., headache and dizziness) were rare among Hungarian vapers, although some previous studies indicated more common occurrence [16–18, 27]. Cardiovascular AEs such as heart palpitation and chest pain were also infrequent especially among e-cigarette-only users compared to other studies [16, 17, 20]. A possible mechanism of neurological and cardiovascular symptoms is short-term increase of heart rate and blood pressure following e-cigarette use, however findings on blood pressure changes after vaping were inconsistent in previous studies [2]. Additionally, learning period of puffing behavior with a newer generation e-cigarette could result in a nicotine boost and possibly excessive nicotine delivery may leads to increased heart rate and chest pain due to myocardial hypoxia [2, 41]. Other AEs were sporadic and even less frequent than reported in previous studies [16, 17].

This study provides more insights to patterns of perceived beneficial and adverse health effects of vaping, however, limitations exist. First, self-reported data are prone to recall and social desirability bias, particularly past tobacco smoking habits and experiencing AEs and physiological changes. Second, individuals with more positive perceptions and experiences of vaping may have been more motivated to participate in the survey leading to respondent bias. Third, the cross-sectional design and convenience sample limit causal inference. Fourth, we cannot separate the impact of positive expectancies for vaping or a placebo effect from improvements due to reducing combustible cigarette use or quitting smoking. Furthermore, we cannot exclude also the possibilities of a general response tendency toward the improvement in functions in self-report due to cognitive dissonance or other self-servicing biases. The possibility of these effects is reflected in relatively high correlations between factors of perceived health improvement. Sixth, like prior research, we did not compare perceived health impacts of switching to e-cigarettes to complete tobacco use abstinence which would be the optimal strategy to reduce tobacco-induced harm. We also do not have data on the long-term stability of the perceived benefits. Seventh, perceived improvement in health is influenced by several factors that we may did not measure which can explain the relatively low explained variance of health improvement factors. Further research should investigate those factors that can influence how e-cigarette users perceive and report health benefits from their product use. Finally, this study similar to others, is based on a convenience sample of users, therefore the generalization of results is limited, however having a representative sample of e-cigarette users is difficult to define, and rarely applied in e-cigarette research. Furthermore, the pattern of e-cigarette use is continuously changing which may also limit the generalizability of our results.

## Conclusions

In conclusion, our study supports that the majority of e-cigarette-only users report more perceived beneficial changes in physiological functions while less AEs than dual users. However, perceived short-term benefits of e-cigarette use may reinforce users despite the uncertainty of long-term health consequences. Further research needs to determine the role of placebo effects and expectancies on objectively measured health improvements associated with switching from combustible cigarettes to e-cigarettes. Health professionals should provide balanced information regarding the possible short- and long-term health effects of e-cigarettes during consultations with patients. E-cigarettes are not without negative health consequences as numerous minor and some major adverse events have been reported previously and in our study [6]. Furthermore, it remains uncertain whether e-cigarettes can serve as an effective cessation aid, while other approved cessation supports already exists, including over-the-counter nicotine replacement and pharmacotherapy options. However, long-term success rates of first-line, evidence-based smoking cessation support remains underutilized and their long-term efficacy is limited [19, 42]. Future studies should measure e-cigarette use patterns and product characteristics that influence perceived and objective health impacts.

## Additional files

Additional file 1: Survey questionnaire. An English language translation of the survey used to study the perceived health effects of vaping among Hungarian adult e-cigarette-only and dual users. (XLSX 14 kb)

Additional file 2: Psychometric analysis. Exploratory factor analysis followed by a confirmatory factor analysis to estimate the measurement model of improvement in health. (DOCX 17 kb)

## Abbreviations

AE: Adverse event
CFA: Confirmatory factor analysis
CFI: Comparative fit index
COPD: Chronic obstructive pulmonary disease
CPD: Cigarettes per day
EFA: Exploratory factor analysis
OR: Odds ratio
RMSEA: Root mean square error approximation
TLI: Tucker-Lewis index
