# Supplementary material for: Perceived health effects of vaping among Hungarian adult e-cigarette-only and dual users: a cross-sectional internet survey
Source: BMC Public Health. 2019 Mar 13;19:302. doi: 10.1186/s12889-019-6629-0 (PMC6417163; doi:10.1186/s12889-019-6629-0)
Supplement: Supplementary file 2 — Psychometric analysis. Exploratory factor analysis followed by a confirmatory factor analysis to estimate the measurement model of improvement in health. (DOCX 17 kb) [file 12889_2019_6629_MOESM2_ESM.docx]

**Additional file 2:** Exploratory and Confirmatory Factor Analyses of perceived health improvements since initiating e-cigarette use

In order to build the measurement model of perceived health improvements, we performed an exploratory factor analysis with WLSMV estimator and Geomin (an oblique) rotation. The three-factor solution was chosen based on eigenvalues, fit indices and interpretability. The factor loadings are presented in Table S1. We also tested this factor structure in the confirmatory factor analysis framework, in which the satisfactory degree of fit requires the comparative fit index (CFI) to be larger than 0.95, Tucker-Lewis index (TLI) to be larger than 0.95, and root mean square error approximation (RMSEA) to be close to 0.05 indicating excellent fit. Since observed indicators were binary variables, the weighted least squares mean and variance adjusted estimation method was used. The three-factor measurement model yielded excellent degree of fit (χ²=142.5 df=32, CFI=0.985, TLI=0.979, RMSEA=0.058, 90%CI [0.048–0.067]). The model and factor loadings are presented in Figure 1. The standardized factor loadings ranged between 0.59 and 0.96, therefore, the measurement model is supported. Factor loadings are presented in Table S1. The second step was to add the structural part of the model which yielded also an excellent fit (χ²=206.6 df=88, CFI=0.980, TLI=0.972, RMSEA=0.038, 90%CI [0.032-0.045]).

**Table S1.** Exploratory and Confirmatory Factor Analyses of perceived health improvements since initiating e-cigarette use

|  | **Exploratory Factor Analysis*** | | | **Confirmatory Factor Analysis**** | | |
| --- | --- | --- | --- | --- | --- | --- |
|  | **Sensory improvement** | **Mental health improvement** | **Physical functioning** | **Sensory improvement** | **Mental health improvement** | **Physical functioning** |
| Sense of smell | **0.89** | -0.01 | 0.07 | **0.95** |  |  |
| Sense of taste | **1.01** | -0.02 | 0.00 | **0.96** |  |  |
| Appetite | 0.01 | **0.99** | **-0.52** |  | **0.59** |  |
| Sexual life | 0.01 | **1.05** | -0.28 |  | **0.84** |  |
| Mood | -0.09 | **0.86** | -0.01 |  | **0.81** |  |
| Memory | -0.22 | **1.05** | -0.28 |  | **0.92** |  |
| Sleeping | -0.01 | **0.53** | 0.26 |  | **0.76** |  |
| Breathing | 0.45 | 0.03 | **0.51** |  |  | **0.89** |
| Physical well-being | 0.19 | 0.04 | **0.74** |  |  | **0.88** |
| Stamina | 0.14 | -0.05 | **0.95** |  |  | **0.93** |
|  | Correlations between factors | | | Correlations between factors | | |
| Mental health improvement | 0.62 |  |  | 0.59 |  |  |
| Physical functioning | 0.46 | 0.73 |  | 0.68 | 0.74 |  |

Note: N=1,039. *: WLSMV estimator with Geomin rotation. **: WLSMV estimator. Standardized factor loadings are presented in the table. Bolded factor loadings are higher than 0.40.
